# A bibliometric analysis on the health behaviors related to mild cognitive impairment

**DOI:** 10.3389/fnagi.2024.1402347

**Published:** 2024-05-03

**Authors:** Liping Xiao, Chunyi Zhou, Shibo Zhang, Yuncui Wang

**Affiliations:** ^1^Department of Information Technology, Jinan University Library, Guangzhou, China; ^2^School of Nursing, Hubei University of Chinese Medicine, Wuhan, China; ^3^Engineering Research Center for Traditional Chinese Medicine Protection Technology and New Product Development for the Elderly Brain Health, Ministry of Education, Wuhan, China; ^4^Hubei Shizhen Laboratory, Wuhan, China

**Keywords:** mild cognitive impairment, health behavior, exercise, bibliometric analysis, cognitive impairment

## Abstract

**Background:**

Mild cognitive impairment (MCI) is commonly defined as a transitional subclinical state between normal aging and dementia. A growing body of research indicates that health behaviors may play a protective role against cognitive decline and could potentially slow down the progression from MCI to dementia. The aim of this study is to conduct a bibliometric analysis of literature focusing on health behaviors and MCI to summarize the factors and evidence regarding the influence of health behaviors on MCI.

**Methods:**

The study performed a bibliometric analysis by retrieving publications from the Science Citation Index and Social Sciences Citation Index sub-databases within the Web of Science Core Collection. Utilizing VOSviewer and CiteSpace software, a total of 2,843 eligible articles underwent co-citation, co-keywords, and clustering analyses. This methodology aimed to investigate the current status, trends, major research questions, and potential future directions within the research domain.

**Results:**

The bibliometric analysis indicates that research on healthy behaviors in individuals with MCI originated in 2002 and experienced rapid growth in 2014, reflecting the increasing global interest in this area. The United States emerged as the primary contributor, accounting for more than one-third of the total scientific output with 982 articles. Journals that published the most articles on MCI-related health behaviors included “Journal of Alzheimer’s Disease,” “Neurobiology of Aging,” “Frontiers in Aging Neuroscience,” and other geriatrics-related journals. High-impact papers identified by VOSviewer predominantly cover concepts related to MCI, such as diagnostic criteria, assessment, and multifactorial interventions. Co-occurrence keyword analysis highlights five research hotspots in health behavior associated with MCI: exercise, diet, risk factors and preventive measures for dementia, cognitive decline-related biomarkers, and clinical trials.

**Conclusion:**

This study provides a comprehensive review of literature on health behavior in individuals with MCI, emphasizing influential documents and journals. It outlines research trends and key focal points, offering valuable insights for researchers to comprehend significant contributions and steer future studies.

## 1 Introduction

With the increasing global trend of population aging, cognitive health issues have emerged as a significant social challenge. In 1997, American researcher Ronald C. Petersen first introduced the concept of mild cognitive impairment (MCI) in a paper titled “Mild Cognitive Impairment: Transition Between Aging and Alzheimer’s Disease (AD).” MCI acts as a precursor stage of AD, characterized by mild memory decline or slight impairment of other cognitive functions while maintaining basic daily life functioning. Since then, MCI has become a widely studied field, with a growing body of related research (Petersen, 2000).

Mild cognitive impairment, as a precursor to cognitive diseases like Alzheimer’s, not only negatively affects an individual’s quality of life but can also progress to more severe cognitive disorders, placing a significant burden on patients and their families. Statistics indicate that the prevalence of MCI among the global population over 65 ranges from 3% to 42%, with an annual progression rate to AD of 8%–25%, which is 10 times higher than in the normal population (Anderson, 2015). Recent estimates from the HRS HCAP study suggest a 22% prevalence of MCI in individuals aged 65 and above (Manly et al., 2022). Extensive research shows that MCI can be effectively prevented and managed through active lifestyles and cognitive training (Qi et al., 2023; Xing et al., 2024). Scientific evidence demonstrates that adopting good living habits and proper nutrition can significantly slow the progression of MCI (Castro et al., 2023). Preventing MCI relies not only on scientific evidence but also on healthy choices and actions in daily life. A growing body of literature provides evidence that improvements in health behaviors can enhance cognitive function, with common healthy behaviors including physical activities, social interactions, quality sleep, and cognitive training (Livingston et al., 2017). In recent years, research on health behavior interventions for the MCI population has gradually become a prominent topic in the fields of health promotion and disease prevention (Farias et al., 2023).

Bibliometric analysis, a popular and rigorous method, has experienced significant growth in recent years in scientific research, providing a powerful tool for the in-depth exploration and analysis of large volumes of scientific data. Discussions on bibliometrics date back to the 1950s, and over time, this method has become increasingly crucial in interpreting and mapping accumulated scientific knowledge and subtle differences in mature fields (Ellegaard and Wallin, 2015). This study aims to utilize bibliometric analysis to investigate the potential impact of health behaviors on MCI prevention. Through a comprehensive bibliometric analysis, we will examine aspects such as the volume of literature, collaboration trends, research collaboration networks, keyword contribution analysis, and citation network analysis to gain a profound understanding of the relationship between health behaviors like exercise, diet, lifestyle habits, and cognitive function. By deeply comprehending the literature in the field of MCI prevention, this study seeks to provide a scientific basis for the development of more effective cognitive health intervention strategies in the future.

## 2 Research methodology

### 2.1 Data source and processing

To ensure the authority and comprehensiveness of the study data, the Web of Science Core Collection (WoSCC) was utilized as the data source, specifically drawing from the Science Citation Index Expanded (SCI-EXPANDED) and Social Sciences Citation Index (SSCI) databases. The rationale behind selecting WoSCC over Scopus was its inclusion of a wide range of influential and high-quality journals from around the globe (You et al., 2021).

When selecting the primary subject term, “mild cognitive impairment” was identified as a clear medical term, with “MCI” serving as its abbreviation. However, upon conducting literature retrieval and analysis, it was discovered that “MCI” had alternative meanings, such as “maximum cardiac index” and “millicurie.” To prevent data interference, the search term for MCI was specified as “mild cognitive impairment.” In terms of determining subject terms related to “health behavior,” using solely “Health Behavior” produced limited search outcomes and failed to encompass all literature on the topic. After reviewing a substantial number of relevant articles (Lee et al., 2010), the subject terms for health behavior were eventually established to include “Health Behavior,” “Lifestyle,” “Physical Activity,” “Exercise,” and “Diet.” The search query was configured as “TS = ‘mild cognitive impairment’ AND TS = (‘Health Behavior’ OR ‘Lifestyle’ OR ‘Physical Activity’ OR ‘Exercise’ OR ‘Diet’).” The publication type was restricted to “article” and “review article,” with no constraints on publication date. The search was executed on 21 November 2023, resulting in the retrieval of a total of 2,843 pertinent articles as the data source for this study. Relevant articles were extracted and downloaded on the same day to prevent bias stemming from frequent database updates. Basic information for each research piece was obtained as “full record and cited references” for further analysis.

### 2.2 Analytical methods and tools

The research methodology employed in this article utilized bibliometric strategies. Two Java-based visualization tools, namely VOSviewer and CiteSpace, were utilized to present the findings from the bibliometric analysis. VOSviewer, developed by the Quantitative Studies group at Leiden University in the Netherlands, is a tool designed for visualizing and analyzing scientific literature. It boasts advanced graphical representation capabilities and can generate knowledge maps depicting units and their relationships within research literature (van Eck and Waltman, 2010).

For the bibliometric analysis of the knowledge base and research trends on health behavior and MCI, this study employed VOSviewer for knowledge mapping analysis, utilizing VOSviewer version 1.6.19. Initially, all records and citation data from the retrieved 2,843 articles were imported into VOSviewer for co-citation analysis, co-word analysis, and cluster analysis. The co-occurrence function of VOSviewer was utilized to investigate keyword associations, with the counting method set to full counting and the unit of analysis focused on author keywords (AKs). Node size variations indicated the strength of the connection effect for each node, while association strength was used to normalize the links’ strength between items (Chen et al., 2022).

**FIGURE 1:**
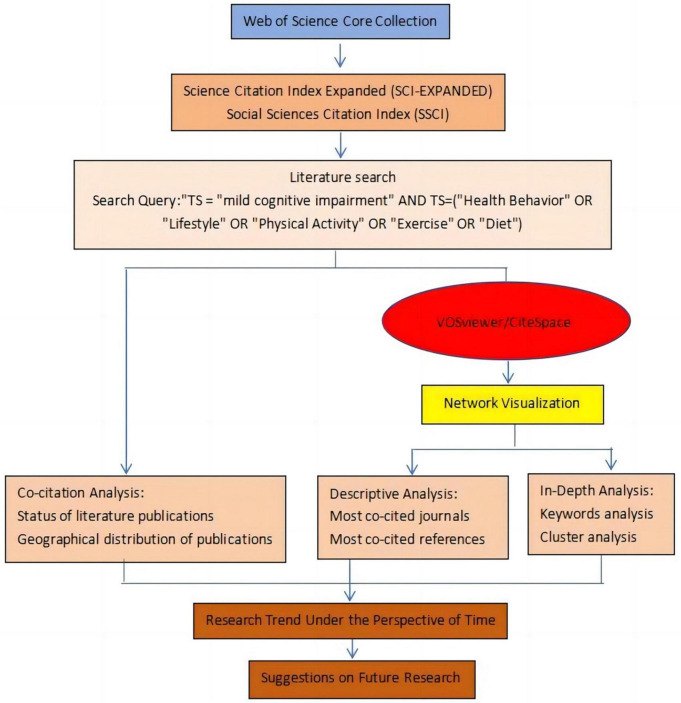
The structure of this review.

The version of CiteSpace used was 6.1R1, which facilitated the creation of a dual-map overlay of journals within the research field. The research structure of this review is depicted in Figure 1.

## 3 Results

### 3.1 Status of literature publications

**FIGURE 2:**
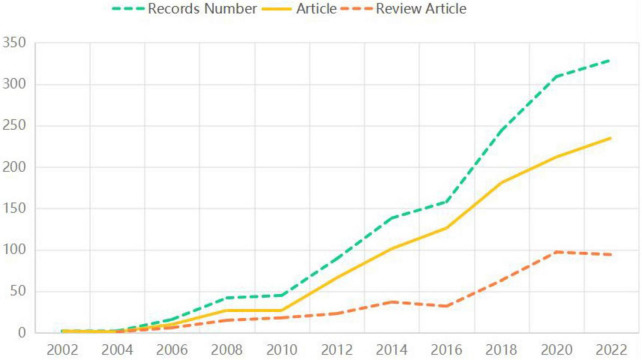
The status of literature publications (2000–2023).

From Figure 2, it is evident that there were no publications before 2002. The first research focusing on the prevention and treatment of MCI through the lens of health behavior emerged in 2002. Subsequently, there was a gradual increase in publications over the years, with a notable surge in 2014. The number of published articles reached 138 in 2014, peaked at 380 in 2021, and has consistently hovered around 300 in both 2022 and 2023. The majority of the research literature consists of articles, comprising 71%, while review articles account for 29%.

These findings suggest that as the global population ages and the prevalence of MCI rises, there is a growing emphasis on enhancing the cognitive wellbeing of older adults. The increasing number of studies in this area aims to develop more comprehensive and sustainable solutions for cognitive health issues in the elderly, ultimately contributing to improved societal health and wellbeing.

### 3.2 Geographical distribution of publications

According to Table 1, research on health behaviors associated with MCI is predominantly concentrated in the top 10 countries or regions, including the United States, China, Australia, England, Canada, among others. Over the past two decades, 84 countries or regions have been actively engaged in researching health behaviors linked to MCI. The United States leads with a total of 982 publications, representing 34.541% of the research output. Following closely is China with 431 articles, accounting for 15.16% of the total publications. Significant contributions have also been made by countries such as Australia, England, and Canada. The top 10 countries collectively contribute to 107.174% of the total publications, indicating a clear trend of concentration. (Due to collaborative research efforts, such as joint publications between the United States and China totaling 82 articles, there is an overlap in counting, resulting in a total percentage of 151.597%.)

**TABLE 1:** The geographical distribution (top 10).

Country/region	Count	Percentage	Country/region	Count	Percentage
United States	982	34.541	Italy	219	7.703
China	431	15.160	Japan	174	6.120
Australia	322	11.326	Germany	173	6.085
England	249	8.758	Spain	166	5.839
Canada	220	7.738	South Korea	111	3.904

**FIGURE 3:**
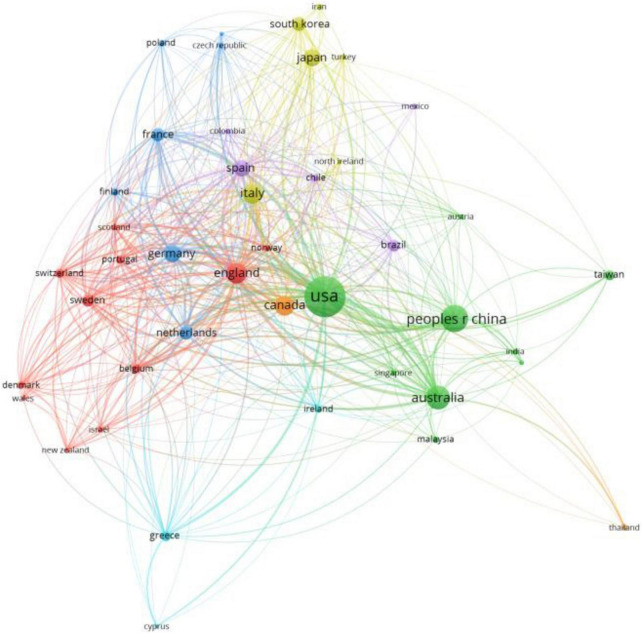
The mapping of countries’ collaboration analysis (2000–2023).

Figure 3 visually illustrates the collaboration patterns among countries or regions in terms of research output through co-authorship analysis conducted using VOSviewer. Countries or regions with more than 10 publications were included, leading to 43 out of 84 entities meeting the criteria. The visualization highlights frequent collaborations between countries or regions, particularly between the United States and China, the United States and Australia, and the United Kingdom and Australia, among others.

**FIGURE 4:**
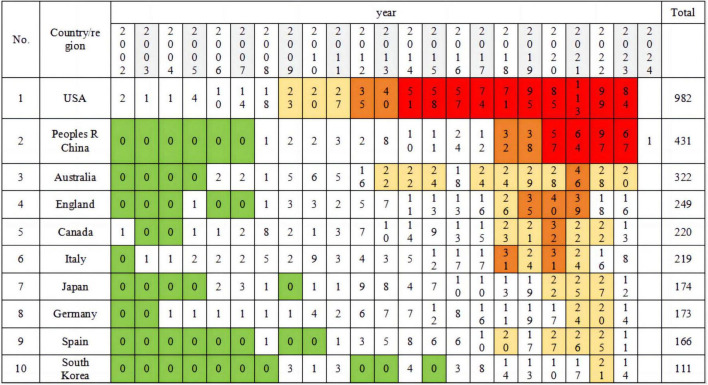
Top 10 country/region and their annual distribution of publications.

As depicted in Figure 4, the annual distribution of research publications concerning MCI and health behavior in the top 10 countries or regions over the past two decades is presented. It is evident that the United States and Canada were among the earliest countries to explore the relationship between health behavior and MCI, with the United States maintaining a leading position in research in this field. China’s research contributions have gained prominence since 2020, with a significant increase in research output observed over the past 3 years from China, Australia, and England.

### 3.3 Most co-cited journals

**FIGURE 5:**
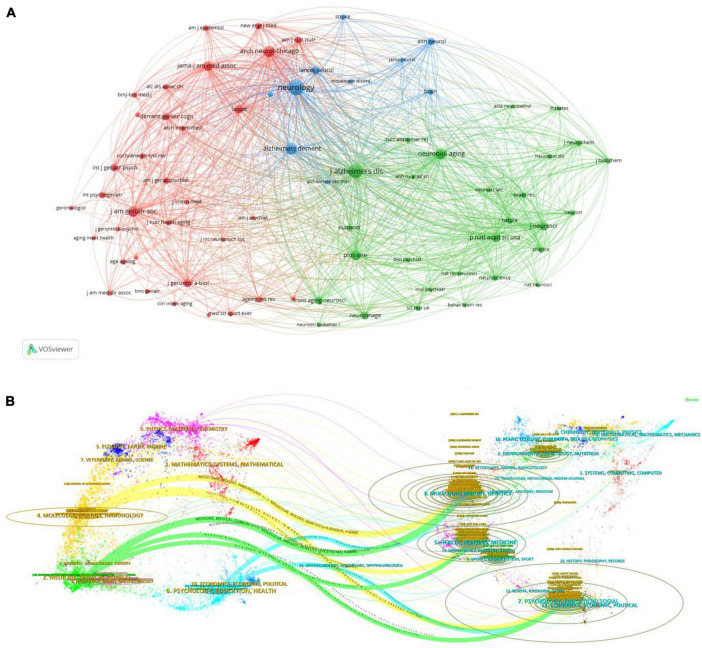
**(A)** The mapping of cited sources co-citation analysis. **(B)** A dual-map overlay of journals.

Figure 5A depicts a visualization of citation references generated by the VOSviewer tool using co-citation analysis. Nodes in the figure represent the sources of literature, while links indicate co-citation relationships between the literature pieces. Literature pieces sharing the same color indicate a close relationship between them. For this study, literature with citation frequencies exceeding 600 times was selected, resulting in a total of 71 literature pieces meeting this criterion, while the dataset consisted of 14,154 cited literature pieces.

Table 2 lists the top 10 cited journals during the study period, ranked by total link strength (TLS). Notably, “Neurology” emerges as highly influential, with both its local citation score (LCS) and TLS significantly surpassing other journals, underscoring its authority in the realm of health behavior and MCI research. Among the top 10 journals, 8 are based in the United States, indicating the concentration of MCI research within the country. The JCR categories of these top 10 cited journals suggest a diverse disciplinary coverage, encompassing Clinical Neurology, Neurosciences, and Geriatrics/Gerontology. These journals have played a pivotal role in advancing research on health behavior and MCI, offering varied perspectives on the subject.

**TABLE 2:** Highly total link strength cited journals (top 10).

Journal	JCR category	Host country	LCS	TLS
Neurology	Clinical Neurology	United States	7,858	452,443
Journal of Alzheimer’s Disease	Neurosciences	Netherlands	6,244	374,292
Neurobiology of Aging	Geriatrics/Gerontology	England	3,797	263,533
Archives of Neurology	Clinical Neurology	United States	4,074	228,398
Alzheimers & Dementia	Clinical Neurology	United States	4,240	226,375
Proceedings of the National Academy of Sciences of the United States of America	Multidisciplinary Sciences	United States	2,629	192,563
PLoS One	Multidisciplinary Sciences	United States	3,213	191,532
Journal of Neuroscience	Neurosciences	United States	2,293	181,407
JAMA – Journal of the American Medical Association	Medicine, General & Internal	United States	2,971	173,509
Journal of the American Geriatrics Society	Geriatrics & Gerontology	United States	3,517	166,125

Similar to Table 2 and Figure 5A also highlights the most influential research categories in the domain of health behavior and MCI. Notably, the green cluster dominated by Geriatrics-related journals such as “Journal of Alzheimer’s Disease,” “Neurobiology of Aging,” and “Frontiers in Aging Neuroscience” is prominently featured. The red cluster comprises interdisciplinary journals like “Archives of Neurology,” “JAMA – Journal of the American Medical Association,” and “Lancet,” while the blue cluster is predominantly composed of neurology journals such as “Neurology” and “Lancet Neurology.” The co-citation relationships among journals in these clusters underscore the interdisciplinary and cross-integrative nature of research on MCI and health behavior.

Figure 5B showcases a dual-map overlay of journals that have published literature related to MCI and health behavior fields using CiteSpace. This visualization method employs two graphs simultaneously, with the left graph representing the categories of citing journals and the right graph indicating the disciplines of cited journals. Citation links reveal the flow of citations within datasets. By juxtaposing citing and cited journals, this map offers insights into the citation relationships between disciplines (You et al., 2021). Three main citation paths are discernible: the yellow path suggests that literature from “Molecular Biology, Immunology” journals tends to cite journals in the “Molecular Biology, Genetics” domain, the green path indicates that articles from “Medicine, Medical, Clinical” journals predominantly cite journals in the “Health, Nursing, Medicine” domain, and the blue path signifies that literature from “Psychology, Education, Health” journals leans toward citing journals in the “Psychology, Education, Social” domain.

### 3.4 Most co-cited references

**FIGURE 6:**
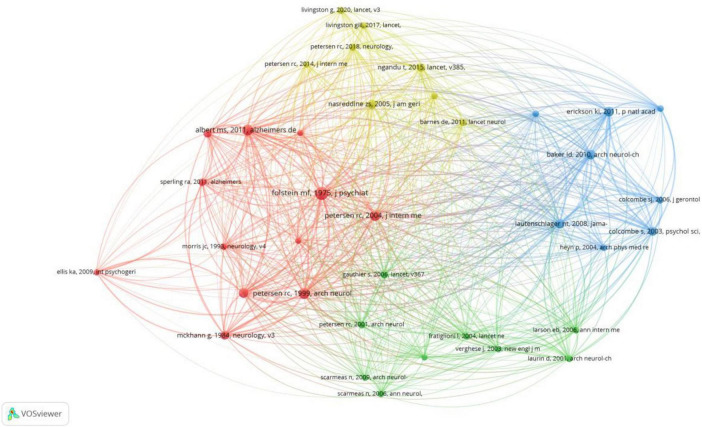
The mapping of cited references co-citation analysis.

Figure 6 displays the outcomes of co-citation analysis of cited references visualized using VOSviewer. Cited literature pieces with local citation scores (LCS) exceeding 100 were chosen, resulting in the identification of 37 cited references from a dataset of 123,962 cited literature pieces. In this visualization, nodes represent the cited references, while links signify the co-citation relationships between them. A consistent color scheme is utilized to denote the close relationships among the cited references.

Table 3 showcases the top 22 highly cited literature pieces identified based on LCS and TLS metrics using VOSviewer within the dataset. These literature pieces span from 1969 to 2020 and predominantly comprise “articles” and “reviews,” sorted by TLS. Additionally, the table presents the Global Citation Score (GCS) calculated by Google Scholar and the LCS calculated by VOSviewer. The GCS reflects the total number of citations of the literature in Google Scholar, whereas the LCS measures the number of citations between literature pieces within the locally retrieved set, offering insights into peer attention within the specific field of research on MCI and health behavior.

**TABLE 3:** Highly total link strength cited references (top 22).

No.	Literature title	Type	Year	LCS	GCS	TLS
1	“Mini-mental state”: A practical method for grading the cognitive state of patients for the clinician	Article	1975	486	105,521	1,373
2	Mild cognitive impairment: Clinical characterization and outcome	Article	1999	317	12,128	1,216
3	The diagnosis of mild cognitive impairment due to Alzheimer’s disease: Recommendations from the National Institute on Aging-Alzheimer’s Association workgroups on diagnostic guidelines for Alzheimer’s disease	Article	2011	308	10,428	1,072
4	Effect of physical activity on cognitive function in older adults at risk for Alzheimer disease	Article	2008	243	2,223	1,053
5	Effects of aerobic exercise on mild cognitive impairment	Article	2010	271	1,506	1,032
6	Mild cognitive impairment as a diagnostic entity	Article	2004	301	8,864	971
7	Exercise training increases size of hippocampus and improves memory	Article	2011	273	5,335	937
8	Mild cognitive impairment – beyond controversies, towards a consensus: report of the International Working Group on Mild Cognitive Impairment	Article	2004	258	5,538	922
9	Fitness effects on the cognitive function of older adults: A meta-analytic study	Article	2003	207	5,358	848
10	The Montreal Cognitive Assessment, MoCA: A brief screening tool for mild cognitive impairment	Article	2005	240	22,285	655
11	Clinical diagnosis of Alzheimer’s disease: Report of the NINCDS-ADRDA Work Group under the auspices of Department of Health and Human Services Task Force on Alzheimer’s Disease	Review	1984	212	33,218	650
12	Aerobic exercise training increases brain volume in aging humans	Article	2006	143	2,833	613
13	A 2 year multidomain intervention of diet, exercise, cognitive training, and vascular risk monitoring versus control to prevent cognitive decline in at-risk elderly people (FINGER): A randomised controlled trial	Article	2015	199	3,043	607
14	The diagnosis of dementia due to Alzheimer’s disease: Recommendations from the National Institute on Aging-Alzheimer’s Association workgroups on diagnostic guidelines for Alzheimer’s disease	Article	2011	178	14,754	589
15	Toward defining the preclinical stages of Alzheimer’s disease: Recommendations from the National Institute on Aging-Alzheimer’s Association workgroups on diagnostic guidelines for Alzheimer’s disease	Article	2011	161	7,613	552
16	Current concepts in mild cognitive impairment	Article	2001	150	6,405	549
17	Exercise is associated with reduced risk for incident dementia among persons 65 years of age and older	Article	2006	117	2,290	540
18	The effects of exercise training on elderly persons with cognitive impairment and dementia: A meta-analysis	Article	2004	125	1,879	510
19	Physical activity and risk of cognitive impairment and dementia in elderly persons	Article	2001	129	2,332	505
20	Aerobic exercise and neurocognitive performance: A meta-analytic review of randomized controlled trials	Review	2010	131	1,979	495
21	Mediterranean diet and mild cognitive impairment	Article	2009	115	933	319
22	Mediterranean diet and risk for Alzheimer’s disease	Article	2006	123	1,379	315

Figure 6 and Table 3 delineate the highly cited papers identified based on the VOSviewer LCS metric, categorizing the knowledge base of research on MCI and health behavior into four primary clusters. These clusters encompass topics such as MCI, diagnostic criteria and assessment, the impact of multifactorial interventions on cognitive function in older adults (including physical exercise, diet, leisure activities, interventions targeting potential risk factors), and more.

The first cluster, focusing on the concept of MCI, diagnostic criteria, and cognitive assessment, is depicted by the red section in Figure 6 and encompasses articles 1, 2, 3, 6, 8, 11, 14, and 15 in Table 3. These articles address various facets of cognitive function, cognitive impairment, and neurodegenerative conditions like AD within the domain of neuroscience. Some literature delves into the concept and diagnostic criteria of early cognitive decline, particularly the discourse surrounding MCI, situated between normal aging and dementia. Conversely, certain studies aim to enhance existing assessment tools, such as the Mini-Mental State (MMS) examination, for a more convenient and effective evaluation of patients’ cognitive status. For instance, Folstein et al. (1975) introduced a simplified cognitive assessment tool, the MMS, which can be administered in just 5–10 min, making it more suitable for elderly patients. Petersen et al. (1999) offer a clinical characterization of patients with MCI, highlighting that MCI patients demonstrate less impairment in cognitive domains other than memory compared to those with AD. McKhann et al. (1984, 2011), Albert et al. (2011), and Sperling et al. (2011), drawing on the most recent revisions of AD diagnostic criteria by the National Institute on Aging and Alzheimer’s Association workgroup, categorize the diagnostic criteria into core clinical and research criteria. They propose the concept of a “preclinical” stage that presents a crucial opportunity for therapeutic interventions for MCI. Additionally, Petersen (2004) and Winblad et al. (2004) discuss the concept and understanding of MCI, addressing controversies surrounding its implementation in diverse clinical settings.

The second cluster highlights the positive impact of physical exercise, specifically aerobic exercise, in safeguarding and enhancing cognitive function in elderly individuals with MCI. In Figure 6, this cluster is denoted in blue and comprises articles 4, 5, 7, 9, 12, 18, and 20 in Table 3. These studies all conducted pertinent randomized controlled trials, with findings indicating that aerobic exercise can reverse hippocampal volume loss in elderly individuals, enhance spatial memory, significantly increase gray matter volume, and improve executive control processes in older adults.

Lautenschlager et al. (2008) demonstrated, through a randomized controlled trial, that 6 months of physical exercise had a moderate positive impact on cognition in elderly individuals with MCI, with effects persisting for up to 18 months. Colcombe and Kramer (2003), Heyn et al. (2004), and Smith et al. (2010), utilizing randomized controlled trials and meta-analyses, discovered that aerobic exercise can enhance executive control processes in older adults, particularly attention and processing speed. Moreover, physical exercise has been shown to enhance physical fitness, bodily functions, cognitive function, and behavior in dementia patients. Aerobic exercise is also linked to improvements in executive function, attention, processing speed, and memory.

The third cluster, highlighted in green in Figure 6, delves into the role of diet, leisure activities, and other factors in preventing cognitive impairment and dementia. This cluster encompasses articles 16, 17, 19, 21, and 22 in Table 3. Collectively, these studies underscore the significance of early detection of cognitive impairment, the impact of lifestyle elements like exercise, diet, and leisure activities on cognitive wellbeing, and the necessity of global research endeavors to comprehend and prevent cognitive decline.

Scarmeas et al. (2009), in a prospective study involving 469 elderly individuals, discovered that engaging in leisure activities such as reading, playing board games, musical instruments, and dancing was linked to a reduced risk of dementia in older adults. Additionally, Scarmeas et al. (2006) explored the correlation between the Mediterranean diet (MeDi) and MCI and cognitive decline. The findings suggest that high adherence to the MeDi is associated with a decreased risk of MCI and the progression from MCI to AD.

The fourth cluster concentrates on the effects of multifactorial interventions on cognitive function in older adults, primarily targeting modifiable risk factors for cognitive decline and impairment, including monitoring vascular risks, diabetes, hypertension, obesity, depression, among others. Represented in yellow in Figure 6, this cluster includes articles 10 and 13 in Table 3. Ngandu et al. (2015), through the randomized controlled trial of the Finnish Geriatric Intervention Study to Prevent Cognitive Impairment and Disability (FINGER), suggests that multifaceted interventions such as cognitive training and monitoring vascular risks could potentially enhance or sustain cognitive function in older adults at risk.

### 3.5 Emerging themes from the literature

In this study, we employed VOSviewer to perform a co-occurrence analysis of author keywords extracted from 2,843 articles focusing on research related to MCI and health behavior. The aim of this analysis was to pinpoint research hotspots and trends within this domain. The connections between keywords indicate the strength of their co-occurrence, with thicker lines denoting higher co-occurrence strength and shorter lines indicating closer relationships. Keywords that are closely related are depicted in the same color.

Prior to the analysis, the data underwent a cleaning and term merging process using VOSviewer. This merging process involved consolidating keywords with similar meanings and standardizing different spellings of words that convey the same meaning. For instance, various terms related to Alzheimer’s disease, such as “alzheimer-disease,” “alzheimers-disease,” “alzheimer disease,” and “alzheimer’s,” were standardized to “alzheimer’s disease.” Similarly, phrases associated with Parkinson’s disease, such as “parkinsons-disease” and “parkinsons disease,” were merged into “parkinson’s disease.” The process of term merging and data cleaning is essential for maintaining term consistency and ensuring accurate analysis.

**FIGURE 7:**
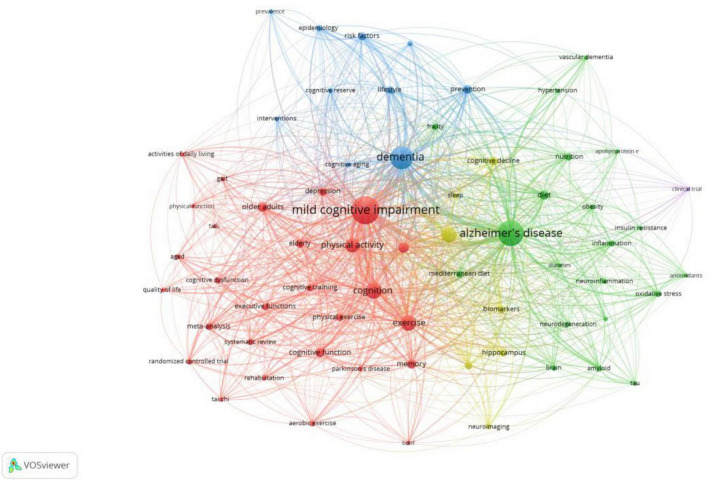
The mapping of author keywords co-occurrence analysis.

Subsequently, a co-occurrence frequency analysis of author keywords was conducted in VOSviewer, focusing on keywords with frequencies exceeding 20. Out of the 4,491 keywords in the dataset, 66 keywords met these criteria. The co-occurrence analysis of author keywords in the realm of MCI and health behavior research is illustrated in Figure 7, highlighting five clusters of different colors. Table 4 provides a summary of the primary research concepts and key topics within the five clusters identified through the co-occurrence analysis of author keywords in this field.

**TABLE 4:** Research concepts and hot topics.

Cluster	Concept	Nodes (*n* = 67)
1	Exercise	Cognition, exercise, mild cognitive impairment, memory, older adults, cognitive impairment, cognitive function, depression, meta-analysis, elderly, physical exercise, physical activity, systematic review, cognitive training, executive functions, cognitive dysfunction, randomized controlled trial, Parkinson’s disease, aged, aerobic exercise, Tai Chi, gait, activities of daily living, quality of life, physical function, BDNF, rehabilitation, and falls (*n* = 28)
2	Alzheimer’s disease	Diet, nutrition, Mediterranean diet, oxidative stress, amyloid, inflammation, frailty, vascular dementia, Alzheimer’s disease, brain, obesity, tau, neurodegeneration, diabetes, neuroinflammation, insulin, hypertension, antioxidants, apolipoprotein E, and insulin resistance (*n* = 20)
3	Dementia	Prevention, risk factors, lifestyle, dementia, epidemiology, subjective cognitive decline, cognitive reserve, interventions, cognitive aging, and prevalence (*n* = 10)
4	Aging	Biomarkers, cognitive decline, aging, hippocampus, magnetic resonance imaging, neuroimaging, and sleep (*n* = 7)
5	Clinical trial	Clinical trial (*n* = 1)

The background colors are primarily used to highlight the publication trends of countries or regions more clearly. Yellow, orange, and red respectively represent the number of documents published by a country or region exceeding 20, 30-40, and over 50 in the current year.

## 4 Discussion

This study has conducted a comprehensive bibliometric analysis focusing on the preventive and therapeutic roles of healthy behaviors in MCI. Our findings reveal a substantial increase in related publications since 2002, with a significant surge post-2014, indicating the growing global interest in this area. The United States emerges as a leading contributor in this field, accounting for over one-third of the publications, followed by China, Australia, England, and Canada. This geographical distribution underscores the global acknowledgment of healthy behaviors in addressing cognitive decline. Additionally, our study demonstrates a multidisciplinary approach in the literature, encompassing fields such as neurology, geriatrics, and neuroscience, indicating a comprehensive understanding of MCI and its intervention strategies. The rise in collaborative efforts, especially between the United States and China, underscores the importance of international cooperation in tackling this public health issue. These insights lay the foundation for future research directions and stress the necessity for a united global endeavor in exploring and implementing effective interventions for MCI.

Having established the increasing global interest and multidisciplinary nature of research on healthy behaviors and MCI, we now transition to examining the five key research hotspots that have emerged. These focal areas not only signify the most significant trends in the field but also showcase the diverse approaches and theories being explored by researchers worldwide. The co-occurrence frequency analysis in this study has broadly classified the themes into five clusters: exercise, Alzheimer’s disease, dementia, aging, and clinical trials. This information offers valuable insights into the current status and trajectory of research concerning MCI and healthy behaviors, unveiling the prominent topics in the realm of MCI and health behavior research.

The first cluster in our study, “Exercise,” comprises 28 keywords, with “Exercise” and “Mild Cognitive Impairment” as the key terms, supported by secondary keywords like “physical activity” and “older adults.” Researchers have conducted extensive randomized controlled trials and meta-analyses, particularly focusing on cognitive function training in the elderly, especially individuals aged 60 and above. These studies have shown that structured, purposeful physical activities and exercises, especially aerobic exercises and physical labor, play a significant role in assisting and safeguarding against MCI in older populations. Meta-analyses from various sources suggest that among all comprehensive or single-component interventions, multimodal exercise combined with cognitive training demonstrates the most significant improvement in overall cognitive function (Xue D. et al., 2023). The research indicates that mind-body interventions have the strongest supporting evidence (Veronese et al., 2023), and multi-component exercises provide significant benefits in alleviating depression in older adults with MCI (Liu et al., 2023).

The second cluster, Cluster 2 (green), primarily focuses on diet, inflammation, and Alzheimer’s disease, incorporating 20 keywords such as “diet,” “nutrition,” “Mediterranean diet,” “oxidative stress,” “amyloid,” and “inflammation.” Studies have emphasized the role of gut microbiota in neuroinflammation and synaptic dysfunction, particularly in AD. Dysbiosis in the gut microbiota can result in gut products like amyloid and lipopolysaccharides (LPS) entering the circulatory system and affecting the central nervous system, thereby influencing brain-related cognitive functions (Bairamian et al., 2022). Diet can modulate the composition of the gut microbiota, serving as a crucial mediator of dietary effects on the host and impacting cognitive functions through the “gut-brain axis” (Cutuli et al., 2023). Current dietary interventions for MCI primarily include the Mediterranean diet (Ballarini et al., 2021), dietary approaches to stop hypertension (DASH) diet (Xu et al., 2018), Mediterranean-DASH Intervention for Neurodegenerative Delay (Hosking et al., 2019), and the ketogenic diet (An et al., 2018).

Cluster 3 (blue) focuses on the factors influencing dementia and intervention measures, encompassing 10 keywords. The National Institutes of Health (NIH) in the United States highlights the active identification of high-evidence-level risk factors for AD to promote primary prevention and reduce its incidence and prevalence (Daviglus et al., 2010). A systematic review and meta-analysis have resulted in the development of the first global evidence-based guidelines for AD prevention (Yu et al., 2020). These guidelines provide level I recommendations for ten factors/interventions, including maintaining a healthy body mass index, engaging in stimulating mental activities, adopting a healthy lifestyle to prevent diabetes, and preventing head trauma. Research supports that a multifactorial intervention approach can enhance cognitive functions, incorporating physical exercise and homocysteine-lowering treatment (Chen et al., 2024). Sustaining a healthy lifestyle, which includes healthy dietary habits, regular physical activity, active social involvement, engaging in positive cognitive activities, avoiding smoking or quitting smoking, and refraining from alcohol consumption, can postpone memory decline, even in the presence of genetic risk factors like the APOE ε4 genotype (Jia et al., 2023). Furthermore, traditional Chinese medicine and acupuncture are gaining recognition (Yee et al., 2018; Xue H. et al., 2023), with studies indicating that practices such as Tai Chi (Jasim et al., 2023; Xue H. et al., 2023; Chen et al., 2024), acupoint massage (Sun et al., 2021), and other traditional Chinese health behaviors can effectively deter cognitive decline.

Cluster 4 (yellow) focuses on biomarkers associated with cognitive decline, incorporating seven key terms. Researchers, utilizing biomarkers and advanced neuroimaging techniques such as magnetic resonance imaging (MRI) and neuroimaging, are dedicated to comprehending the biological, neurological, and behavioral mechanisms through which healthy behaviors mitigate MCI (Sørensen et al., 2017). The analysis of serum biomarkers, particularly those associated with neuroinflammation, oxidative stress, and metabolism, offers biological indicators of cognitive decline (De Felice and Lourenco, 2015). In recent years, researchers have started employing new methods, like machine learning algorithms, to investigate biomarkers for AD, enabling the quantitative evaluation of the effectiveness of healthy behaviors (Xu et al., 2023). Neuroimaging, especially MRI, plays a crucial role in this research. MRI allows researchers to observe changes in brain structure, especially in regions linked to cognitive functions such as the hippocampus and prefrontal cortex (Sorensen et al., 2016; Bast et al., 2017). This is crucial in unveiling the impact of healthy behaviors on brain structure, providing concrete evidence for the enhancement of cognitive function. Additionally, sleep plays a vital role in influencing the connection between health behaviors and MCI (Künstler et al., 2023). Sufficient sleep duration and quality are fundamental for cognitive function and overall brain health (You et al., 2023, 2024). Studies have indicated that sleep disorders and disruptions in circadian rhythms can heighten the risk of developing MCI by facilitating the accumulation of amyloid-beta (Aβ) and tau proteins. This, in turn, raises the risk of cognitive impairments (Barthélemy et al., 2020; Posner et al., 2021). Furthermore, sleep disturbances and deprivation can impair critical cognitive processes in MCI, such as memory consolidation, attention, and executive function (Kong et al., 2023). A study has shown that implementing Cognitive Behavioral Therapy for Insomnia (CBT-I) in elderly individuals with MCI significantly enhances participants’ sleep quality and executive functions in cognition (Cassidy-Eagle et al., 2018). This implies that health behavior interventions targeting sleep could potentially boost cognitive function in elderly individuals with MCI.

Cluster 5 (purple) primarily delves into empirical studies on the impact of healthy behaviors on MCI, with “clinical trial” as its sole keyword. One study employed an integrative intervention approach for MCI patients, incorporating risk factors like healthy lifestyle habits and physical activity into cognitive training (Gong and Tao, 2021). The findings suggested that this socio-psychological comprehensive care model could enhance cognitive function and quality of life in MCI patients. Another study showcased that cognitive-physical dual-task training yields clinical benefits by improving executive function and instrumental activities of daily living in older adults with MCI. Cognitive-physical dual-task training emerges as a promising intervention for older adults with MCI (Kim and Park, 2023). Nonetheless, challenges persist in clinical interventions for the MCI population, such as high participant dropout rates, low overall participation, and short follow-up durations. Future research should prioritize conducting larger-scale, high-quality clinical studies to explore more modifiable risk factors and their association with the onset of MCI, thus presenting promising avenues for MCI prevention.

The analysis from the aforementioned clusters highlights the research trends in the prevention and treatment of MCI through healthy behaviors. Lifestyle factors, including physical activity engagement, sleep quality, and nutritional habits, play a crucial role in the potential prevention of MCI (Dominguez et al., 2021). These factors often interact synergistically, influencing cognitive health and resilience against cognitive decline. Quality sleep is essential for memory consolidation, neuronal repair, and overall brain health, with disruptions in sleep patterns linked to an increased risk of cognitive impairment (Künstler et al., 2023). Similarly, nutrition plays a vital role, with a balanced diet rich in antioxidants, omega-3 fatty acids, and vitamins supporting brain function and reducing inflammation, which are associated with MCI risk (Li et al., 2022). Understanding the intricate interplay between these lifestyle factors and their collective impact on cognitive health is crucial for developing effective preventive strategies against MCI and promoting successful aging.

## 5 Future research direction and limitations of this study

The increasing trend in publications since 2002, particularly the surge post-2014, reflects a growing interest that presents opportunities for further exploration through international collaborations. Collaborations between leading contributors like the United States and emerging researchers from other countries can facilitate a deeper understanding of the factors influencing cognitive function. Future research is likely to place greater emphasis on interdisciplinary collaboration and integrated research methods, drawing on knowledge and techniques from diverse fields such as biology, neurology, geriatrics, psychology, and sociology. This holistic approach aims to comprehensively unravel the preventive and therapeutic effects of healthy behaviors on MCI. Through interdisciplinary collaboration, researchers can delve into the intricate relationship between cognitive function and health behaviors, offering more comprehensive and effective guidance for future interventions and treatment strategies.

Advancements in biomarker research represent another crucial area of focus. By leveraging more sophisticated biomarker analysis and neuroimaging techniques, future studies can enhance our understanding of the biological mechanisms underpinning MCI and provide a more precise assessment of the impact of various healthy behaviors.

However, it is important to acknowledge the limitations of this study. Firstly, the accuracy and reliability of the bibliometric analysis depend on the quality and source of the data, and relying solely on the WoS database may have resulted in the exclusion of relevant studies. Secondly, the focus on English-language articles introduces potential linguistic bias, potentially overlooking valuable research conducted in other languages. Therefore, further exploration across multiple databases and languages is warranted.

Scientometric is an emerging approach that utilizes mathematical and statistical methods to quantitatively and qualitatively analyze research trends and investigate the status of specific topics (Chen et al., 2022). In comparison to traditional systematic reviews or meta-analyses, bibliometric analysis can swiftly summarize and identify trends across a vast number of publications. It visually represents the research landscape using data such as citation counts and network characteristics, illustrating the connections between studies and offering objective measures of influence and relevance. However, there are certain drawbacks to consider. Bibliometric analysis heavily relies on data sources and citation metrics, which can be influenced by publication biases and variations in citation practices among different fields (Weingart, 2005). Unlike systematic reviews, bibliometric analysis typically does not evaluate the quality or content of the research. It provides a macro-level perspective that may overlook nuanced findings and methodological quality (Zupic and Cater, 2015). Additionally, bibliometric analysis might overlook context-specific insights that systematic reviews are better suited to capture. In conclusion, our bibliometric analysis can delineate the scope and trends of research concerning health behaviors in MCI, furnishing more specific information for systematic reviews or meta-analyses.

## 6 Conclusion

This study, employing bibliometric analysis, discovered that research on the healthy behaviors of individuals with MCI originated in 2002 and experienced rapid growth starting in 2014. Over the past two decades, the United States has emerged as the leading country in terms of publications, contributing 982 articles, representing 34.54% of the total scientific output. Noteworthy journals that have published extensive research on MCI-related healthy behaviors include “Alzheimer’s Disease Journal,” “Journal of Neuroaging,” and “Frontiers in Geriatric Neuroscience,” alongside other publications focused on gerontology. Co-occurrence keyword analysis identified five research hotspots in MCI healthy behaviors. Future research endeavors could benefit from integrating multidisciplinary perspectives through international collaborations, advancing biomarker research, and employing sophisticated biomarker analysis and neuroimaging techniques to deepen our understanding of the biological mechanisms underlying MCI and to accurately evaluate the impact of healthy behaviors.

## Data availability statement

The original contributions presented in this study are included in this article/supplementary material, further inquiries can be directed to the corresponding author.

## Author contributions

LX: Conceptualization, Data curation, Software, Writing – original draft, Writing – review & editing. CZ: Conceptualization, Writing – review & editing. SZ: Visualization, Writing – review & editing. YW: Funding acquisition, Resources, Supervision, Writing – review & editing.
